# Theoretical psychology: discursive transformations and continuity in Psychological Research/Psychologische Forschung

**DOI:** 10.1007/s00426-022-01727-2

**Published:** 2022-08-27

**Authors:** Alexander Nicolai Wendt, Uwe Wolfradt

**Affiliations:** 1 Department of Human Sciences, Università degli Studi di Verona, Verona, Italy; 2 Department of Psychology, Ruprecht-Karls-Universität Heidelberg, Heidelberg, Germany; 3 Department of Psychology, Martin-Luther-Universität Halle-Wittenberg, Halle, Germany

## Abstract

*Psychological Research* (formerly *Psychologische Forschung*) has been published for a century which makes it a valuable subject matter for historical investigations. The journal’s development bears traces of the progress in psychology. This development is of particular interest for the field of theoretical psychology which investigates the epistemological and methodological background. Our hypothesis is that the history of *Psychological Research* is indicative for the transformations within the discourse of the discipline, i.e., the general context of communication in psychology. We revisit the changes in the editorial practises of the journal through a scientometric mixed-methods approach, combining bibliometric analyses which compare *Psychological Research* to *Psychological Review* and the *British Journal of Psychology* with a single-case investigation. Regarding form, we find continuities and disruptions in the development of the editorial customs from long and single-author to short and multi-author contributions. Investigating content through word frequency analysis shows that the journal’s history reflects the rise of the cognitivist paradigm as well as a transition from theoretical discourse towards experimentation. The analysis of a single case demonstrates the nature of past theoretical discourse in contrast to contemporary practises. Overall, our findings support the assumption of discursive transformations. From the perspective of theoretical psychology, these transformations can be described as a shift towards Methodism which entails a critical negligence of theory.

## A historical appraisal from the standpoint of theoretical psychology

Scripture is the principal medium of science. Embedded in the general context of language, it ascertains the documentation of insights. Yet, the form of scientific language is not stable itself. On the one hand, the gradual change of general language, be it grammatical or semantical, is something like a tectonic drift for scientific speech, on the other, terminology depends on epistemic paradigms which undergo sometimes negligible, sometimes disruptive changes themselves. Scientists who investigate the object of their research interest do not necessarily notice the relevance of these changes when they only use their language instead of reflecting on it. This reflection does not come without effort and conditions. First, it requires methodology, for example linguistic or epistemological. Second, an empirical perspective is necessary that allows for a detachment from contemporary debate. Differently put, one must employ both theory and history of science. In psychology, these are a concern of ‘theoretical psychology’. In this article, we wish to take the standpoint of theoretical psychology to investigate 100 years of scientific scripture as our historical data, namely, the history of the journal *Psychological Research* (formerly *Psychologische Forschung*). From this perspective, theory building’s dependency on historical context becomes visible.

We aim to continue a series of prior investigations about the meta-scientific context of *Psychological Research*. Scheerer (1988), Guss (2019), and Heuer (2021), to mention the primary examples, have undertaken historical analyses of journal’s development. Hence, our approach integrates into history of science, more specifically, history of psychology. A paradigmatic example for this kind of research is Martin Kusch (1998). Kusch analyses the conflicts in the background of the so-called Würzburg School to explain the rise and fall of German thought psychology. Our analysis of *Psychological Research* has a similar outline, which comprises a descriptive and an explanatory goal. First, we wish to propose a description of the general changes that have occurred in the journal’s history. Our focus for it is the role of theory in psychology. Second, we wish to deliberate a possible explanation for this development.

We begin by elaborating on the standpoint of theoretical psychology in the first part. In our interpretation of the term, ‘theoretical psychology’ is neither the sum of all theories in psychology nor an independent sub-discipline that compares to ‘theoretical physics’. In fact, theoretical psychology is an aspect of psychological science which is embodied in any form of research to a higher or lesser degree, be it explicit or implicit. Concretely speaking, in the widest sense of the word theoretical psychology is the epistemological, methodological, and historical discourse which underlies empirical investigation. Take, for example, a contemporary experimental study which searches for neural correlates of behavior. This investigation incorporates a subtext which encompasses theoretical positions, such as cognitivism or psychophysics whose epistemological constitution reaches back in disciplinary history to contributions made by classics, such as Edward Titchener or Wilhelm Wundt. It is not necessary for the contemporary text itself to address these connections. This is only true for theoretical psychology in the narrower sense of the word.

Based on the standpoint of theoretical psychology, the second part of our article will describe and interpret the history of *Psychological Research* by a scientometric investigation. Our method of choice are bibliometrics. At this point, the bibliometric self-description of the journal has a tradition of its own. We attempt to complement the previous meta-scientific publications on *Psychological Research* with our focus on theoretical psychology. The center of our investigation is what we call ‘discursive transformations’. In the bibliometric data, we expect to find evidence for the development of form and content of psychological discourse in the 100 years of research at hand.

The subject of discursive transformations cannot be exhausted by quantitative analyses alone. Therefore, the last part of this article will attempt qualitative descriptions of a paradigmatic individual case within a series of publications that represents the discursive transformations of the journal. Methodologically, the utility of single cases has been questioned, especially concerning generalizability (Donmoyer, 2000; Hurtado-Parrado & López-López, 2015). However, the investigation of a single case has a specific purpose in the given context. We do not only wish to assess superficial shifts like the change from German to English language which has been made a standard for publications in *Psychological Research* in 1974. Investigating a single case within the history of science calls for close reading as a method to expose the details of publication practises. James Collier and David Toomey (1997, especially chapter 2) present an approach to this form of inquiry into the details of scientific text. They draw on Ivor Richards’ conceptual groundwork of close reading to formulate a systematic guide for the confrontation with scientific texts. Close reading is, like other approaches, a technique that attempts to attain the highest level of description. Such close reading of individual texts is a standard form of research within the field of history of psychology (e.g., Hajek, 2015; Kendler, 2020).

Our research interest concerns the very nature of psychological discourse: What is the direction that our discipline, for which the journal can claim to stand like few others, has taken in the last century? In this vein, we focus on an issue that has proven to be of greatest relevance in the last decade due to, for example, the meta-discursive phenomenon which has been called “replication crisis” (e.g., Fiedler & Prager, 2018; Open Science Collaboration, 2015). This issue is the role of theory in psychology. We claim that theoretical discourse—which should not be confused with theoretical psychology—has lost relevance over the course of the last decade. Hence, psychology has lost its discursive resilience that could help with overcoming phenomena like the ‘replication crisis’. However, inter-disciplinarity, especially between psychology and philosophy, would need to play a bigger role to regain this resilience: Looking at past discursive transformations naturally leads to ideas for the new ones.

### What is theoretical psychology?

Psychology is usually conceptualised as a positive science (cf. Carson, 2012). This means that it inherits the legacy of a positivist emancipation from philosophical patronage. The psychological approach to insight stands in the tradition of critical rationalism, logical empiricism, empirio-criticism, and ultimately empiricism as a worldview that has been systematized since early modernity by classics, such as Galileo Galilei, Francis Bacon, or John Locke. As a consequence, there are two primary methodological parts of contemporary experimental psychological research insofar as it stands in a positivistic tradition: Applied mathematics and empirical data (for a discussion see: Bickhard, 2001; Michell, 2003). Philosophy of science, on the other hand, is not understood as a core issue of the discipline but instead as a meta-disciplinary concern which is pursued by philosophy as *ancilla scientiae*, a notion that has become relevant over the course of the emergence of natural science as well as the priority of epistemology in nineteenth century philosophy (Beiser, 2014).

Is this conception of psychology as a science self-evident and uncontroversial? Certainly not. Quite on the contrary, the self-description of psychology as a scientific endeavor has seen many deviations from the smooth path of positivism. To some degree, it might even be justified to return to Karl Bühler’s description of the situation within psychology as a “structural crisis” (Bühler, 1926): A crisis that is not transitional but inherent to the discipline *itself*. And even more critically, the conception of science in general as a positivist project has received major critique throughout the twentieth century, for example in the ‘positivism dispute’ (cf. Strubenhoff, 2018). Thus, it is not safe to say that the methodology of empirical psychology is ‘positive’ or ‘exact’ (cf. Galliker, 2016). Instead, it is a question for theory of science and this question concerns psychologists themselves because it is not only a question *about* psychology but *within* it.

Theoretical psychology can be considered as a meta-psychology which strives to reach a systematic summary of the mental facts and to find a clarification of the lawful relations between them. Lindworsky (1932, 2/3) establishes four points as the main concerns of theoretical psychology:

1. Classification of the countless empirical facts by virtue of a comprehensive system.
2. Reducing these facts to a relatively small number of basic facts and assumptions.
3. Deduction of not yet observable phenomena from the established theoretical propositions.
4. Stimulation of new experiments and observations, which in turn corroborate the theoretical conception.

In contrast to experimental psychology, theoretical psychology strives to grasp psychological problems from a holistic point of view, i.e., its focus does not lie on individual facts but on the structure and relations of individual facts. According to Lindworsky, theoretical psychology, tries to uncover laws that describe the connection between psychological phenomena and organise them into meaning units (see Wolfradt, 2012).

In accordance with Fahrenberg, we describe theoretical psychology as “systematics of controversies” (Fahrenberg, 2015). In the wider sense of the term, this means that all psychological investigations are imbued with a subtext of methodological and epistemological problems whose influence is independent of its explication. To add an example: Any empirical investigation faces the problem of metrization, be it implicit or explicit. However, those works that do not explicate the problem tend to perpetuate the issue and might even contribute to the loss of understanding. In other words, without explicitly facing methodological and epistemological problems, they will not be solved and might cause unknown harm to the superordinate discursive context.

Whenever psychologists engage with problems of methodology, epistemology, ontology etc., they do ‘theoretical psychology’ in the narrower sense. It means validating the presuppositions of research instead of relying on them. However, this does not require an independent sub-discipline of psychology. On the contrary, almost all investigations conduct theoretical psychology to some degree. To give an example, one could say that the parts of scientific articles which are usually labelled ‘introduction’ and ‘discussion’ are at least partly dedicated to theoretical psychology. Whoever reflects on the scope of research, the historical background, or possible methodological limitations engages with ideas which exceed the thematic constraints of empirical investigation. Yet, pursuing ‘theoretical psychology’ in the narrow sense of the word calls for a systematic approach. It means more than, for example, discussing issues like empirical limitations due to sample size at the end of an investigation. In fact, theoretical psychology is a field of research whose methodology is currently underdeveloped because it is not ‘positive science’ but requires reflection and historical discourse.

The reasons for the neglect of theoretical psychology are manifold. As can be claimed from the standpoint of phenomenological hermeneutics (Kockelmans, 1993) or critical theory (cf. Frisby, 1972; Gadenne, 1972), the positivist conception of science as a data-driven marketplace of ideas is a predominant concept that is characteristic for critical rationalism. It was Reichenbach (1938) who distinguished the context of discovery and the context of justification. Whereas the latter is a question of logical reasoning, the prior is a seldom branch of science which relies on scientific intuition and creativity: Where do theories come from? It is an unhandy question for the (Kantian) traditions of criticism. Ultimately, the inability to tackle it effectively has led to its extrusion from methodology (Yet, the later Popper returned to the issue when investigating the nature of ‘problems’; see Popper, 1992). Hence, the utmost relevance of theoretical psychology for theory-building has been overlooked due to the dominance of classical critical rationalism in psychological methodology.

Another reason for the demise of theoretical psychology is that it is reliant on inter-disciplinarity (Teo, 2019). It requires references to specific self-referential disciplines, such as philosophy or sociology of knowledge and science, to establish a meta-scientific perspective. Yet, the interdisciplinary orientation of psychology has shifted away from these disciplines and opened itself towards biology and computer sciences, instead. While these contributions can also be valuable, they do not provide a methodology for a meta-scientific outlook. As a matter of fact, these disciplines do not share the same epistemological background. For example, they are oblivious of the mind–body problem. Discursive transformations like this have led to a relative ignorance of the ubiquity of theoretical controversies in the fabric of psychology.

### Theoretical psychology and theory in Psychological Research

One might argue that, in spite of the alleged relevance of theoretical psychology for the discipline, it must have a place or a discourse of its own which cannot be identical with publications and publication organs that report on empirical findings in the first place. Even though this argument is valid because theoretical psychology in a narrower sense is not identical with all psychological work, it requires a historical point of view to understand the implications of this division of labor in research. Discursive transformations are also transformations of scientific culture. As the two following sections will show, the history of *Psychological Research* is entwined with theoretical psychology in a far-reaching manner.

To give but one example: Guss (2019, 93–98) enlists the 26 entries long series of publications called ‘contributions to the psychology of shape’ (*Beiträge zur Psychologie der Gestalt*) which were mainly published in *Psychological Research* (but also in other contexts) between 1913 and 1937, representing a vivid and long-lasting exchange of empirical and theoretical ideas that develop around the notion of *Gestalt*. What ties these contributions together is a mutual theoretical background and the journal as a platform of conversation. This example shows that theoretical psychology has been inherent to the publication customs of the journal’s past. Hence, one cannot simply say that *Psychological Research* was not the place to engage with theoretical psychology.

Nowadays, extensive discussions which overarch decades and include several scientific protagonists are still possible, but it is less likely to encounter them in the same format as in the early twentieth century. One obvious reason is that the conceptual adherence to schools of thought, such as the Berlin school of psychology of shape (*Gestaltpsychologie*), has become less typical for our discipline. Another is that the discipline has grown substantially so that the alignment of the discourse in accordance with single individuals like the famous trio of Kurt Koffka, Wolfgang Köhler, and Max Wertheimer is less likely. One way or another, the descriptive point of reference remains the same: The nature of the psychological discourse as well as of the discipline’s culture have changed and the development of *Psychological Research* is an expression of this change. These considerations bring us to the guiding question for our investigation: Has the function of theory and theoretical science changed over the course of the discipline’s history, given the 100 years of *Psychological Research*? And if so, what are these transformations?

A possible explanation for changes in the practices of research over last century is the tendency of specialisation, or, differently put, disintegration. In general philosophy of science, this type of transformation within sciences has been described by Kuhn (1982) as a localisation of research which leads to incommensurability. This theory finds recognition and critique in the recent debate of sociology and philosophy of science (Politi, 2019). In order to describe the mechanisms and patterns of specialisation, sociology of science has developed different bibliometric measures (Nicolaisen & Frandsen, 2015; Wichmann Matthiessen & Winkel Schwarz, 1999). We will follow this direction of methodology.

Traces of the tendency towards specialisation can also be found in *Psychological Research*. The journal’s first decades did not only see extensive theoretical discussions of psychological findings, the articles also used to consider implications for other fields of psychology and even other disciplines—*Psychological Research* has originally been an interdisciplinary journal, publishing ethnological and even zoological articles. This tendency of integration is opposed to the contemporary focus on individual findings. In order to describe this discursive trajectory from mereological perspective, we employ the distinction of holism vs. particularism. Holism takes the entirety of a phenomenon or concept into account while mereological particularism (which is conceptually distinct from *conceptual* particularism, see below) extracts an element for closer examination.

The discursive transition from holism to mereological particularism in psychology is correlated with other tendencies. For example, sociology finds a tendency of functionalization in science (Luhmann, 1990). This means that the advancement of science complicates the integration of the field because the expansion of research means additional complexity which must be consequently reduced by decreasing the scope of individual contributions. This compensation of complexity is similar to the division of labor in society (see Sil, 2000). Another parallel trajectory in the history of psychology is the succession of methodological paradigms, especially behaviourism and cognitivism in the second half of the twentieth century. Since both stand in the tradition of American pragmatism, their respective relation to theory also prefigures its role in publications. In this context, it is safe to say that the continental traditions of science favor theoretical psychology more than pragmatism does (for a discussion of this context see Roeber, 2018).

The description of discursive transformations and their explanation naturally leads to a normative evaluation. However, it is a question of scientific rigor to separate these two parts. The following investigation into the history of *Psychological Research* does not aim to judge the development. It serves as an assessment of the status of theory in the discipline. However, no research interest can be motivated without an initial problematization. Hence, we will revisit the meaning of the transformations we describe in the third section.

## A bibliometric investigation of discursive transformations

The quintessence of the first section has been our hypothesis that there has been a discursive transformation in the last century of the discipline’s history which is reflected in the publications of *Psychological Research*. More concretely, our hypothesis is that a main feature of the transformation is a shift from holism to mereological particularism which specifically relates to the relevance of theory within the psychological discourse. In other words, we claim that the role of theory has diminished in one way or another, be it continuously or disruptively, be it by ‘functionalization’ of the discourse or by negligence in the predominant research paradigms.

It is an epistemological question which kind of methodology is serviceable for the investigation of structural shifts in the history of scientific disciplines. Since the perspective of our investigation is meta-scientific, the subject matter cannot be investigated on the object level of the research process. Instead, the research design requires self-referentiality. It calls for philosophy, history, sociology, and ultimately psychology of science. Fahrenberg (2015, 614–682) provides a systematic review of scientometric methodologies, offering several options, such as content analysis, multimethod investigation of reception history, citation frequency, ranking of eminence, bibliometric investigation of trends and dynamics in interests, or cycle of ideas.

Among these alternatives, the bibliometric analysis of trends is most suitable to represent discursive transformations. An example is a frequency analysis of ‘flagship publications’ for different psychological schools over the course of the second half of the twentieth century (Robins et al., 1999). The authors attempted to reflect the shift in relevance of cognitive, behavioural, psychoanalytic, and neuroscientific approaches within psychology by comparing the frequency of respective keywords in four prestigious journals between 1950 and 1999.

Our bibliometric approach entails that the available data are the articles which have been published in *Psychological Research* over the course of the last century as well as the respective bibliographic information. In the context of empirical meta-science, bibliometrics are a standard paradigm which has an elaborate methodology (Glänzel et al., 2019). More specifically speaking, ‘advanced bibliometrics’ (van Raan, 2019) are suitable for our purpose. We make this choice for two reasons. First, we stand in the tradition of the other investigations which have been conducted about Psychological Research before (Guss, 2019; Heuer, 2021; Scheerer, 1988). Second, bibliometrics allow the abstraction from individual contents by means of summary which can render superseding tendencies visible and guide the following investigation of individual cases in the following.

Herbert Heuer’s (2021) recent publication about the history of the journal is an important reference for our own bibliometric investigation. Heuer has reported and interpreted, among other statistics, the page count per article, the impact factor, the authors’ nationalities, the citations of other journals in *Psychological Research* and of *Psychological Research* in other journals. Heuer’s bibliometric investigation gives a concise overview of the general development of the journal which does not contradict our own perspective. On the contrary, we wish to complement it with a more specific research interest. While Heuer successfully describes the development of the journal, we wish to interpret it from the meta-scientific standpoint of theoretical psychology. In this sense, we draw on Heuer’s results but intend to move to a different point of view, namely the investigation of discursive transformations.

In the following, we will make use of different bibliometric data for the scientometric description of discursive transformations. We begin by frequency analyses to provide a general profile of the journal, and then move towards key word analyses, similarly to the procedure described by Fahrenberg (2015, 630–633). As mentioned before, the weakness of keyword analyses is that the meaning of terminology can shift even if the same words are used. For this reason, content analyses ought to complement bibliometric investigations. They can provide a more detailed look after identifying general trends.

Scientometric analyses of this kind do not serve the purpose of binary hypothesis testing, as it is the methodological standard for operationalist psychology (Vessonen, 2021). Due to its self-referential nature, meta-science cannot work towards incremental evidence-based progress (cf. Schoepflin & Glänzel, 2001). Making reference to the sociological discourse, but also being relevant for its psychological counterpart, Gläser and Laudel state: “The classic search for causal explanations that is realised by quantitative sociological methods plays only a minor role in scientometrics: hypotheses are not tested, nor are statistically significant associations that contribute to sociological explanations pursued” (Gläser & Laudel, 2001, 431). Instead, scientometric methods “contribute to comprehending the dynamics of doxographic history” (Fahrenberg, 2015, 683; translation by the authors). Hence, empirical research in the field of scientometrics does not pertain to natural science in the strict sense.

### Developments regarding form

The general purpose of the following quantitative statistics is to establish a descriptive basis which allows evaluating whether and how *Psychological Research’s* publication practice can be characterised as continuous or disruptive. While the bibliometric data in Scheerer (1988) and Guss (2019) highlight the pre-war period of the journal (although Scheerer also separately regards later publications), Heuer (2021) focuses on recent developments. This is reasonable because the journal’s form has changed over the course of its existence. This is already evident in consideration of the most prominent events like the gradual shift from German to English with the official rebranding in 1974 or the period of vacancy during and after World War II. More fine-grained changes are the introduction of the position of editor-in-chief (as well in 1974) or the regular publication of abstracts since the early 1960s as a means of standardization.

Nonetheless, some bibliometrics can be calculated for the entirety of the journal’s history. These bibliometrics are resilient against the changes because they are basic properties of publication. Examples for this kind of statistics are the most frequent authors (Table 1), the most cited articles (Table 2), or the most cited authors (Table 3). They serve the purpose of giving a general overview about the journal’s context. For example, the most frequently quoted text of the journal’s history, Max Wertheimer’s *Studies concerning the Theory of Shape*, stems from the Gestaltist period. It shows that the past status of being the primary outlet for research of shape is still relevant in the present. It must be mentioned that, in spite of being available and meaningful statistics, these data are also subject to cross-temporal changes in publication practise, such as the requirements for admission (e.g., peer review). Also, they do not serve well to depict discursive transformations because of their limited representation of developments. Therefore, the source for robust longitudinal formal data is not as rich as it might appear on first sight. We wish to focus on two aspects in the following.

**Table 1:** Most frequent authors in Psychological Research

Author	Number of texts
Bernhard Hommel	49
Wolfgang Prinz	27
Carlo Umilta	21
Robert W. Proctor	20
Andrea Kiesel	19
Herbert Heuer	19
Iring Koch	17
Nachshon Meiran	17
Hubert D. Zimmer	16
Hans-Christoph Nuerk	14
Wilfried Kunde	14

Data source https://www.crossref.org, September 2021

**Table 2:** Most quoted authors in Psychological Research

Author	Number of citations
Bernhard Hommel	3477
Max Wertheimer	1465
Robert W. Proctor	856
Wolfgang Prinz	796
Iring Koch	690
Carlo Umilta	687
D. Alan Allport	617
Donald T. Stuss & Michael P. Alexander	566
Kim-Phuong L. Vu	558
Hubert D. Zimmer	557

Data source https://www.crossref.org, September 2021

**Table 3:** Most quoted articles in Psychological Research

Title	Authors	Number of citations
Studies concerning the Theory of Shape (1923)	Max Wertheimer	1285
Executive functions and the frontal lobes: a conceptual view (2000)	Donald T. Stuss & Michael P. Alexander	566
Studies concerning Action and Affect Psychology, III. The Retaining of completed and uncompleted Actions (1927)	Bluma Zeigarnik	547
Two mechanisms of vision in primates (1968)	Colwyn Trevarthen	460
A feature-integration account of sequential effects in the Simon task (2004)	Bernhard Hommel, Robert W. Proctor, & Kim-Phuong L. Vu	457
About the effect of domain formation in the track field (1933)	Hedwig von Restorff	449
Task switching and the measurement of "switch costs" (2000)	Glenn Wylie & D. Alan Allport	419
Induced Motion (1929)	Karl Duncker	360
Phonemic deficits in developmental dyslexia (1981)	Margaret J. Snowling	349
The attentional blink: Resource depletion or temporary loss of control? (2005)	Vincent Di Lollo, Jun-ichiro Kawahara, S.M. Shahab Ghorashi, & James T. Enns	332

Data source https://www.crossref.org, September 2021

All data for this investigation stem either from web-scraping with a self-made web crawler, which is an automated digital processing of internet sources for the extraction of information, or from scientific databases, especially Crossref (https://www.crossref.org) and Web of Science (https://www.webofscience.com). We have compiled our data in our own database by means of a self-made program written for the project (with the programming language Matlab; www.mathworks.com/). Our raw data are available in the supplementary material. The data analysis has also been performed with Matlab.

In addition to the bibliometric data about *Psychological Research*, we have included two further journals as an international point of reference, namely Psychological Review and the British Journal of Psychology. The choice was made following four criteria. First, the journals must be as old or older than *Psychological Research*. Second, they must stem from different national backgrounds within the Western psychological discourse. Third, they must have been made object of at least one of the investigations about *Psychological Research* which precede ours. Fourth, they must be sufficiently relevant for the overall development of the discipline. We assessed this fourth criterion based on standard publication indexes, such as the Quartile’s score index as an impact factor. These journals are *Psychological Review*, founded in 1894, one of the oldest journals of the American Psychological Association with a pragmatist tradition, and the British Journal of Psychology, which has been published on behalf of the British Psychological Society since 1904. Both journals have been mentioned by Scheerer (1988) and Heuer (2021), fall in the Q1 quartile of journals in the category Psychology (miscellaneous), were founded before *Psychological Research*, and represent a different cultural background.

**Fig. 1:**
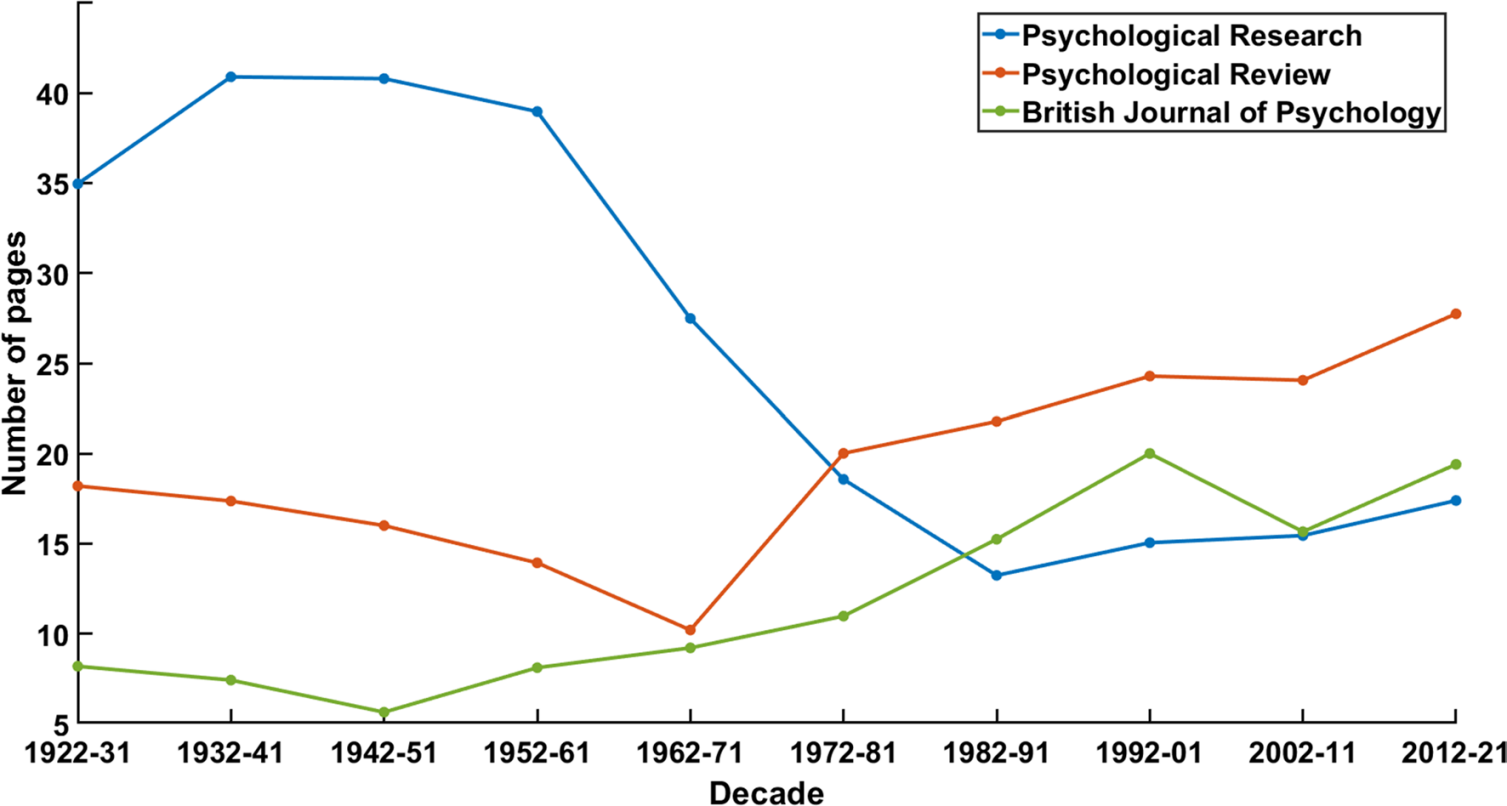
Average article length per decade

Our first bibliometric description of the journal’s history concerns the average article length (see Fig. 1). It has also been reported by Heuer, but only for the period 1990–2020. Heuer highlights “the target length of 8 pages that Engelkamp (2001) mentioned” (Heuer, 2021, 6) and reasons that the recent increase in length might be the result of review practise as well as an increase in relevant literature over time.

However, taking a look into the past shows that the first half of a century of publications in *Psychological Research* did not meet this parsimony. On the contrary, the first four decades (which encompass the break between 1938 and 1949) show an average length of more than thirty pages per article. A phase of transition is visible in the 1960s and 1970s. Heuer considers the 1950s until 1980s a phase of “comeback”, but without continuity since “there was no clear profile of the Journal in the post-war period” (Heuer, 2021, 4). The following years are labelled “consolidation” insofar as different editors-in-chief reformed the publication strategy by ideas, such as Scheerer’s concept of ‘Thematic Issues’. Hence, the “major changes” (ibid.) of these periods reflect in the data, showing a deviation from the publication patterns of the early years. Speaking of a “comeback” in the sense of continuity would partially be justified for the 1950s whereas the 1960s already show signs of “consolidation”.

*Psychological Research*’s development of the average article length over the last century is dissimilar from the two references. The articles published in *Psychological Review* had less than half of the average article length of respective texts published in *Psychological Research*, the *British Journal of Psychology*’s texts even had less than a quarter of the length until the 1950s. The second part of the relevant period sees the different journals’ tendencies converge concerning article length converge. Generally speaking, the texts have become longer in the last decades. However, the articles published in Psychological Review were almost twice as long than the publications in the other two journals since the 1980s.

**Fig. 2:**
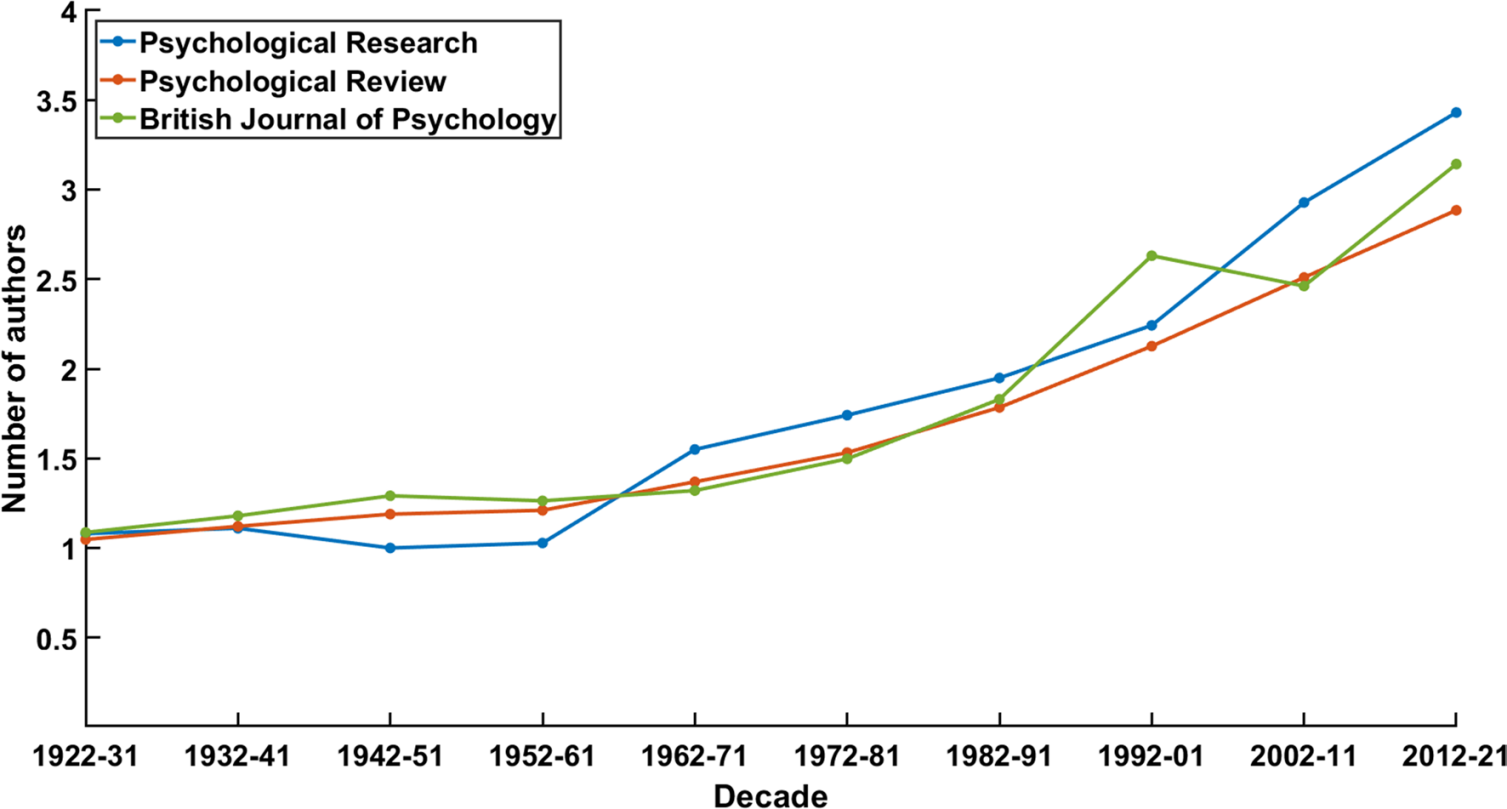
Average numbers of authors per article in every decade

The second statistic about the basic bibliometric data confirms the assumption of discontinuity (see Fig. 2). It depicts the average number of authors per article. Just as the average article length has decreased since the 1960s, the average number of authors has begun to increase in that decade. While the first four decades had customary single-author publications, two- or multiple-author publications have become the norm afterwards. However, while the downward trend of average article length has been slightly inverted since the 1990s, the average number of authors did not cease to increase. Indeed, these two facts might be connected since it seems logical that additional authors also require representation in the texts. Differently put and indirectly agreeing with Heuer (2021), the increase in average article length does not relate to a return to the early publication practise of the journal but is the result of formal changes in contemporary editorial routines.

The comparison with the two reference journals shows convergence since the beginning of the record. The publications in all three journals were authored by a single person in most of the cases for the first half of the relevant time frame. The following increase is almost monotonous in all cases as well, showing a similar acceleration of the curve, even though *Psychological Research* has had the highest average count of participating authors in the last decade. This development begs the conclusion that certain customs and conventions about the nature of psychological research have developed in parallel for different scientific traditions but examining this kind of conjecture requires an intercultural point of view.

It would be speculation to connect these findings with our topic of theory in psychology. At this point, they primarily reflect the existence of global discontinuities and local continuities in the history of *Psychological Research*. Article length and author number are formal aspects of research that are the expression of social conventions which need more specific analyses to be understood. Nonetheless, in the light of more complex and elaborate findings of the following section, we can revisit the aforementioned formal changes in the context of discursive transformations.

### Developments regarding content

A rudimentary formal analysis gives first evidence for the existence of transformations that reach beyond minor oscillations. The fact that in the first half of the journal’s history, most articles had more than two dozen pages and were written by a single author, clearly contrasts with the second half when articles became shorter, less than a dozen pages on average, and were usually submitted by two or a group of authors. The question arises whether these changes remain to be superficial and simply reflect new customs of editorial practise or rather correlate to more substantial shifts in the scope and purpose of the journal—a question that ultimately relates to the classic problem of form and content: Has the change in content solicited new forms? Do the new forms inherit paradigmatic transformations of content? Or can scientific contents of all sorts be expressed by single-authored long or multi-authored short articles?

In order to delve into the depth of psychological content, the bibliometric analysis has to be elaborated. To find an adequate representation of content, we chose to investigate semantic shifts in the journal’s history. This becomes possible on a larger scale thanks to computational technology. The obvious benefit of word frequency analyses is displaying the prevalence of keywords which can be described as ‘operational terms’, a notion in the philosophy of Eugen Fink (1957) that contrasts with ‘thematic terms’ which are the ideas and concepts that are explicitly discussed in science. ‘Operational terms’ on the other hand are the words and thoughts with and through which scientists describe and explain their subject matters without reflecting on them themselves.

Take the example of ‘experiment’. The word is often used to describe the standard procedures of empirical psychology but in most cases, it remains meta-language which does not become the object of the respective investigation: Most experimental psychologists do not seek to understand the essence of experimentation when they experiment, they simply use experiments to understand their field of interest. Still, they use the terms in their publications. Word frequency analyses can try to track the customary use of ‘operational’ and ‘thematic’ notions. In a way, the prevalence of certain terms from scientific meta-language expresses the self-conception of research processes.

What word frequency analyses cannot track are the semantic shifts themselves: Do we mean the same when we say ‘cognition’ as William James did over a hundred years ago? Since it is even doubtful whether contemporaries understand the same ideas when using identical words, the cross-temporal continuity of concepts must remain uncertain. In fact, this problem is a conundrum in the heart of hermeneutics which occupies philosophy of language as well as linguistics. Though it limits the validity and scope of word frequency analyses, it does not extirpate it because it is reasonable to assume a basic semantic continuity. Otherwise, scientific communication would not be possible. Hence, this methodological constraint forms more of a theoretical threshold than a critical limitation. It can be subject to individual, for example etymological, investigations. But this is not the place for them because the resolution of analysis targets the bigger frame of reference.

Our first word frequency analysis was devoted to grasping the paradigmatic development of psychology. It is well known that, in its first decades, *Psychological Research* (as *Psychologische Forschung*) has been the primary outlet of psychology of shape, viz. *Gestalt*. Since this field of research is mainly concerned with perceptual phenomena, one would expect the presence of terminology with reference perception in the early years. The first question that arises is whether this field of research has lost its relevance in the later decades, due to the emergence of cognitive sciences and cognitivism. The second question is whether these movements have left an impact on the journal.

In order to tackle these two questions, we summarised the titles of the 2546 available articles in *Psychological Research* (excluding the book reviews in the first volumes). Titles of scientific research have a clear function of expressing the core content. It can therefore be expected that this most representative part of any text displays fundamental affiliation to a research paradigm. We then examined all 167 words with a frequency of at least 20 mentions. Among these, we selected all terms that have a (certainly not undisputed) connotation of either cognitive psychology or psychology of perception. A similar method for the description of development in the history of science in general and psychology in particular has been used by Halvor Teigen (2002) who investigated the usage of the term ‘law’ over the course of the twentieth century.

**Fig. 3:**
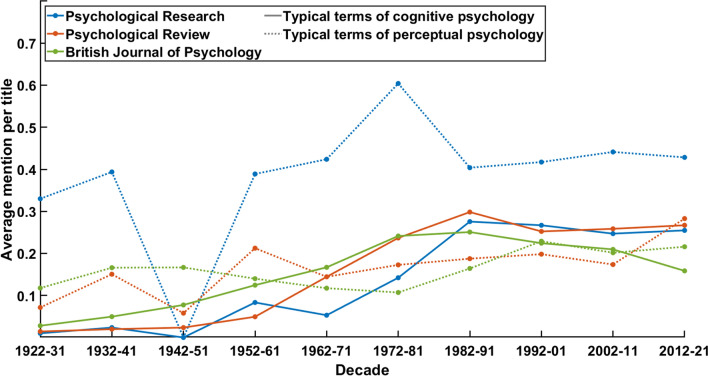
Average mention of typical terms from either cognitive (solid line) or perceptual psychology (dotted line) in article titles per decade. The typical terms of cognitive psychology are displayed with a solid line, they include “representation”, “representations”, “recognition”, “memory”, “cognition”, “knowledge”, “information”, and “cognitive”. The typical terms of perceptual psychology are displayed with a dotted line, they include “visual”, “vision”, “spatial”, “space”, “perceptual”, “perception”, “perceived”, “movements”, “movement”, “motion”, “eye”, “distance”, “color”, and “auditory”

The results (see Fig. 3) show manifest statistical tendencies. First, vocabulary which is indicative for investigations of perception has been in use with about 0.3 to 0.4 mentions per article title in the first decades until the editorial break (which reflects in the drop in frequency in the third decade since only few publications fall into this period). However, the terminology does not lose prevalence in the decades which have been identified as ‘consolidation’, i.e., the 1960s until the 1990s. It does not seem obviously erroneous to interpret these findings as a reason for discursive continuity insofar as *Psychological Research* remains to be an outlet that is open for perceptual psychology. This interpretation does not contradict the self-declaration of the journal’s research scope which remarks independence “of any particular approach or school of thought” (see https://www.springer.com/journal/426), but it highlights a tradition of research.

At the same time, vocabulary which is indicative of cognitive psychology does not show a similar representation. Until the 1960s, this terminology found mention with less than 0.1 mention per title. During the consolidation phase, the frequency rose to 0.2 to 0.3 mentions. This development can be seen as a clear indication of a discursive transformation. The ‘consolidation’ phase has not only been a shift towards new editorial norms but also a development in content. Concretely speaking, the portfolio of *Psychological Research* has been diversified in this phase, adding cognitive psychology to the traditional focus on perceptual psychology. A finding that should not be surprising since this transformation has pervaded the entire discipline as the so-called ‘cognitive turn’. Nonetheless, it is remarkable that this transformation’s expression in article titles did not replace the previously predominant presence of the topic of perception.

The comparative analysis shows that the articles in both, *Psychological Review* (4748 articles) and the *British Journal of Psychology* (5838 articles), have a lower frequency of terms that are indicative of perceptual psychology throughout the century. This difference is evidence for the profile of *Psychological Research* as a journal with a traditional focus on perceptual psychology. While *Psychological Review* averages about 0.4 mentions per title, the two other journals do not deviate substantially from an average of 0.2 mentions. However, the increase of the usage of terms which relate to cognitive psychology takes place about a decade earlier in *Psychological Review* and the *British Journal of Psychology.* This invites the conclusion that the discursive transformation of the cognitive turn has affected the publication customs of *Psychological Research* later than similar primarily anglophone journals. A subsequent historical hypothesis is whether continental or German psychology has followed the cognitive turn that has taken place originally in the English-speaking world. A corresponding analysis can be found in Métraux (1985).

The comparison between the journals begs the question whether the development of *Psychological Research* can be generalised or differs from the others. More specifically, the historical primacy of perceptual over cognitive psychology entails the question whether the advent of cognitive terminology can be predicted by a decline of perceptual terminology. Yet, historical data are not experimental which engenders that the given material does not permit conclusions concerning causality. Nevertheless, a regression analysis can help visualise trends. Since the word frequencies of both domains are correlated to a sufficient degree (*Psychological Research*: *r*(8) = 0.47, Psychological Review: *r*(8) = 0.65, British Journal of Psychology: *r*(8) = 0.24), an exploratory interpretation of a regression model can help understanding whether the development of *Psychological Research* is rather generalizable or unique.

**Fig. 4:**
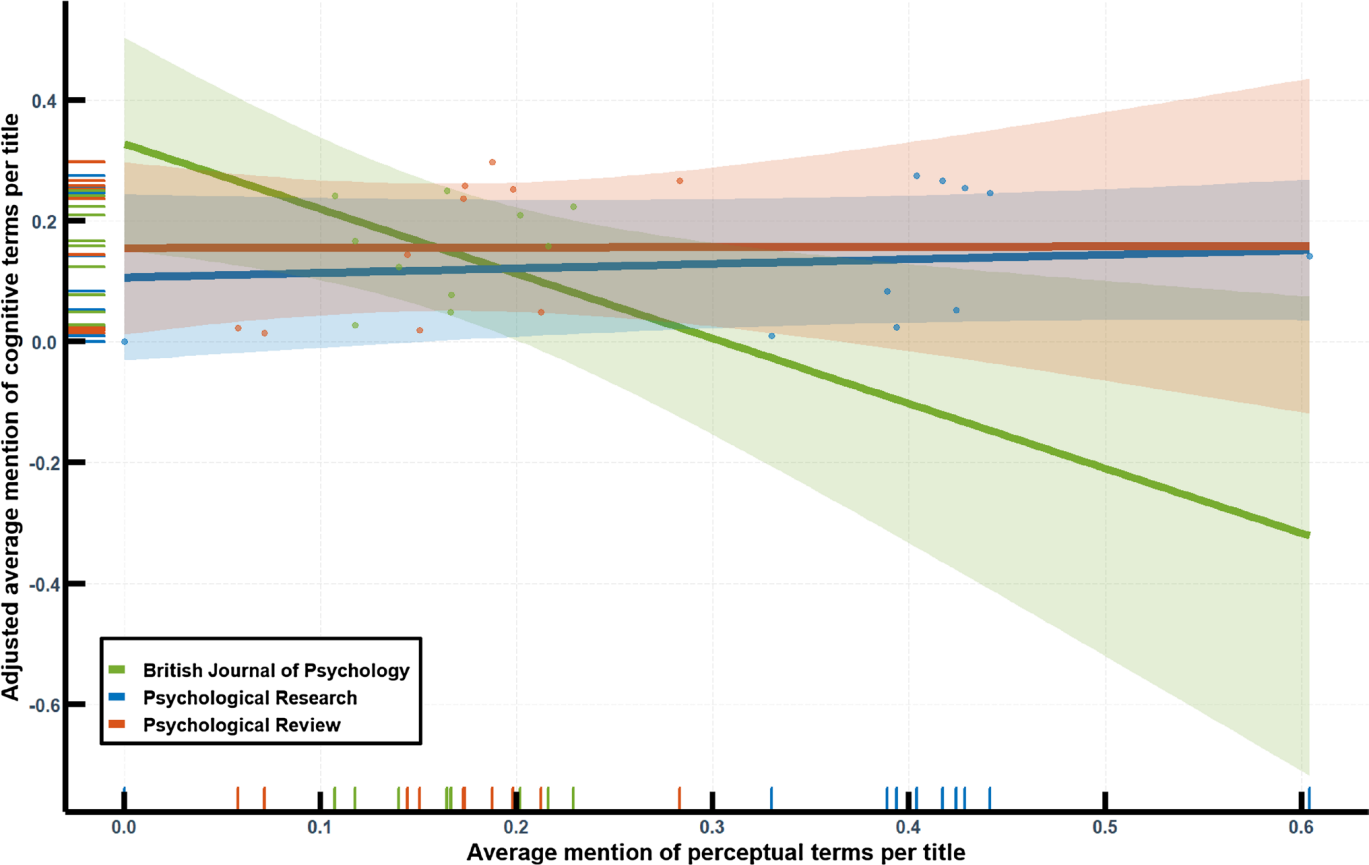
Interaction between perceptual terminology and journal in a regression analysis of cognitive vocabulary. The diagram includes 95% prediction intervals, a scatter plot of the (adjusted) data points, as well as a rug plot to increase visibility of the data

The model predicts word frequency of cognitive vocabulary based on the main effects of time, journal, and frequency of perceptual vocabulary, as well as the interaction of the journal and the perceptual vocabulary. It explains a sufficient portion of variance, *R*^2^ = 0.82, *F*(6,23) = 18, *p* < 0.001. Figure 4 shows that the utilization of cognitive vocabulary was mostly unaffected by perceptual vocabulary, be it in Psychological Review or *Psychological Research*. The development of terminology in the British Journal of Psychology shows a minor difference. In its case, the relation between the two conceptual domains indicates an inverse proportion. However, this finding is marginal due to the minor correlation for the data in the case of the journal. Although all parameters of the model are significant—except the interaction of effect of Psychological Review and the perceptual vocabulary (with *Psychological Research* as reference category)— time is the strongest predictor for the transformation. Thus, it remains clear that the ‘cognitive turn’ has had a similar bearing on all journals under consideration, namely benefitting cognitive vocabulary without replacing or superseding perceptual vocabulary (whose semantics might have changed in the process). In spite of this general similarity, *Psychological Research* differs from the other outlets due to a continuous high relevance of perceptual terminology.

**Fig. 5:**
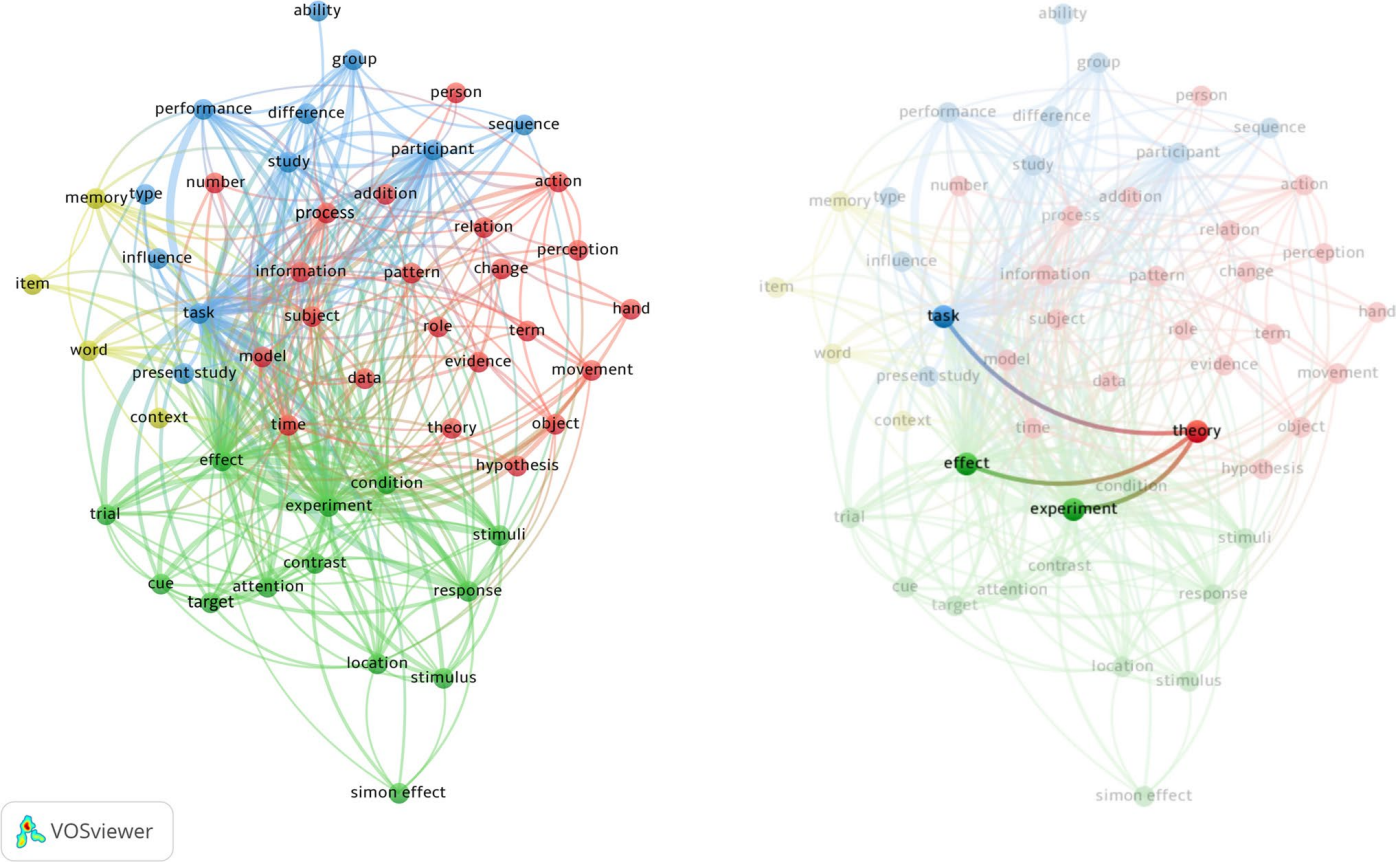
Term map of a cluster analysis based on co-occurrence for the text of all available abstracts in Psychological Research (translation from German or French by an automatic translation API). Right side: All significant links for the term ‘theory’

But how does the conceptual shift in psychological vocabulary affect the primary topic of this article, namely theory in psychology? This question requires a closer look because the usage of ‘operational terms’ in titles seems to be less likely than in the manuscripts themselves because titles usually address the topic of an investigation. ‘Operational terms’ however are used when scientists speak about their topic. In order to further represent the ‘operational’ vocabulary of *Psychological Research*, we decided to examine all abstracts of the available articles that have been published since 1962 (2272 articles) since the standard procedure of using abstracts was adopted by the journal in the early 1960s. Differently put, our data incorporated the relevant publications from the ‘consolidation’ phase and afterwards. A term map of words used in these abstracts gives a first impression (Fig. 5). The term map was created with the VOSviewer software (van Eck & Waltman, 2013).

The map shows the 50 terms which have been mentioned more than 150 times and all links with a minimal strength of 250. The map is clustered by association strength and contains four clusters: The term with the highest link strength in the first cluster (blue) is ‘task’. A rational examination of this cluster suggests that it combines terms which describe the formal background. The second cluster (red) is less homogeneous. The terms ‘time’, ‘process’, and ‘information’ have the highest link strength for this cluster. What connects most of the terms is that they refer to the experimental setup. The third cluster (green) contains the terms ‘effect’ and ‘experiment’ which are strongly linked across the map, but other terms in the cluster indicate that it summarises words which refer to experimental content. The fourth cluster only contains four words, among which ‘memory’ has the highest link strength. It includes specific vocabulary that represents a class of empirical investigations which are less perceptual than the main scope of the journal.

The term ‘theory’ has 444occurences and links to three further terms, ‘task’, ‘effect’, and ‘experiment’ which are the three strongest links for the entire map. Overall, the term theory has a comparably low link strength which ranges in the lower third of all terms. This suggests that reference to theory is generally scarce since the 1960s. Furthermore, the term ‘theory’ does not systematically couple with other notions which would reflect theoretical debate.

Additionally, the abstracts allow a more detailed examination. We employed the same methodology as in the first word frequency investigation, selecting the 537 words with a frequency above 50 as vocabulary for either experimentation or theory. The relevant keywords for theory are all idiomatically related to the term theory itself since theoretical discourse does not usually have instrumental terminology like experimentation and the cluster analysis suggests that no further terms systematically relate to theory.

**Fig. 6:**
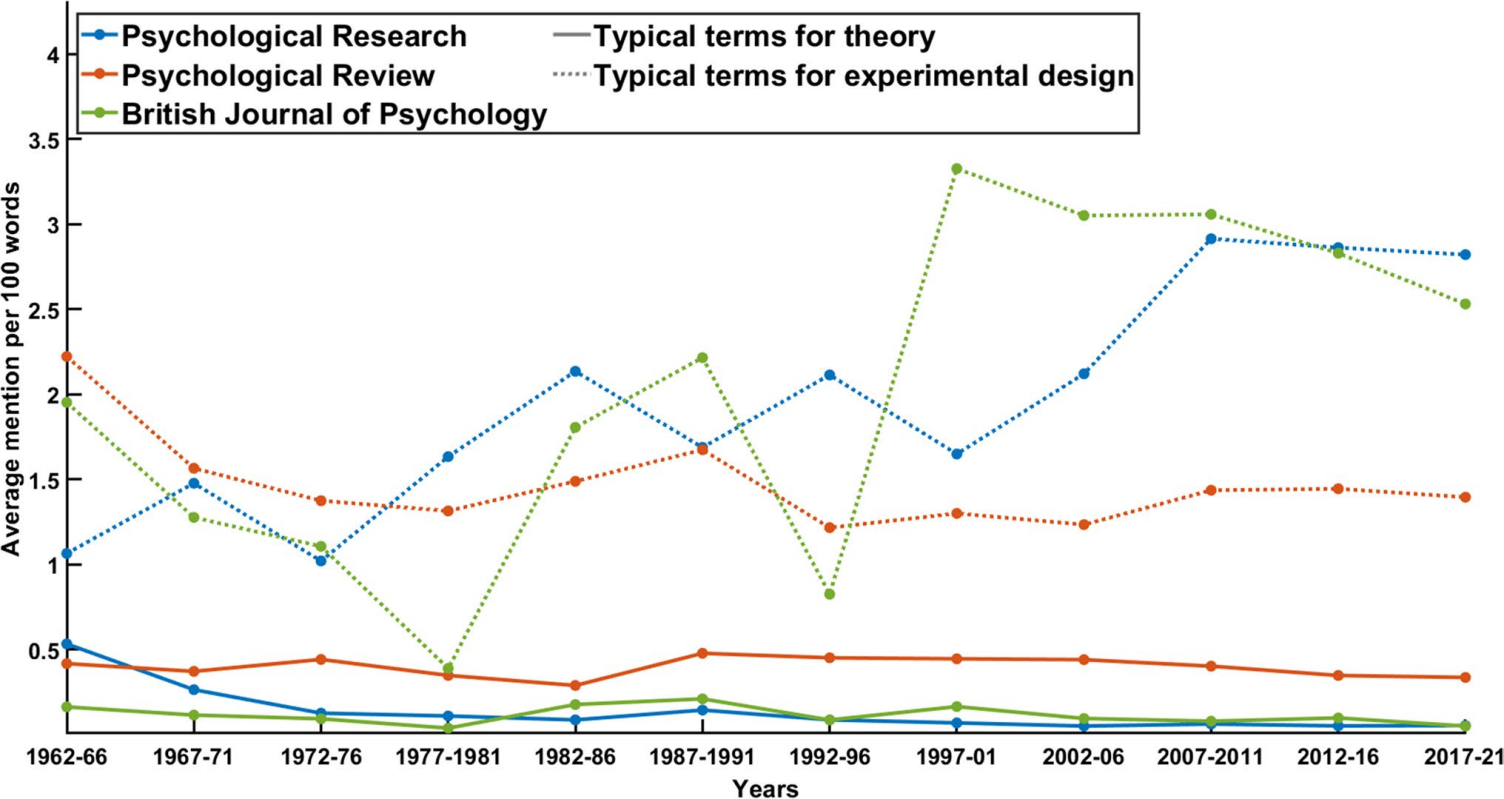
Average mention of typical terms for the theoretical and the experimental discourse in 100 words of abstract over a course of five years. Typical terms for theory were “theoretical”, “theories”, and “theory”. Typical terms for experimentation were “condition”, “conditions”, “control”, “data”, “demonstrate”, “demonstrated”, “effect”, “effects”, “evidence”, “experiment”, “experimental”, “experiments”, “hypothesis”, “instructions”, “items”, “manipulated”, “measure”, “measured”, “measures”, “participants”, “predicted”, “predictions”, “result”, “results”, “task”, and “tasks”

The results (see Fig. 6) give a clear impression of the developments. Keywords for experimentation have been present with increasing frequency in the ‘consolidation’ phase and even more frequent afterwards, reaching more than three mentions per 100 words in article abstracts in the last 15 years. Keywords for theory, on the other hand, have the highest frequency in the early 1960s, being mentioned about once every 200 words of text in abstracts. Since then, the reference to theory has become scarcer, dropping below one mention every 1,000 words in the last decades. These results demonstrate that the ‘consolidation phase’ of the journal’s history correlates with an increase of attention for experimentation, its technical description, and the interpretation of its results while the prevalence of reflection on theory has shrunken at the same time.

The comparative analysis generates partly coinciding results. While the frequencies in the *British Journal of Psychology* are similar, i.e., showing a low usage of theoretical terms while the frequency of terminology for experimentation increases, Psychological Review has a relatively higher percentage of references to theory, keeping the level of about 0.5 mentions per 100 words for the last 60 years. At the same time, comparably less words for experimental design are used. This result finds a clear explanation in the journal’s agenda: “Psychological Review publishes articles that make important theoretical contributions to any area of scientific psychology, including systematic evaluation of alternative theories” (https://www.apa.org/pubs/journals/rev). Yet, the same level of theoretical vocabulary had been in use during the early 1960s in *Psychological Research*. This indicates that the journal had previously incorporated a greater representation of theory, up to a level of a journal whose purpose is theoretical debate.

**Fig. 7:**
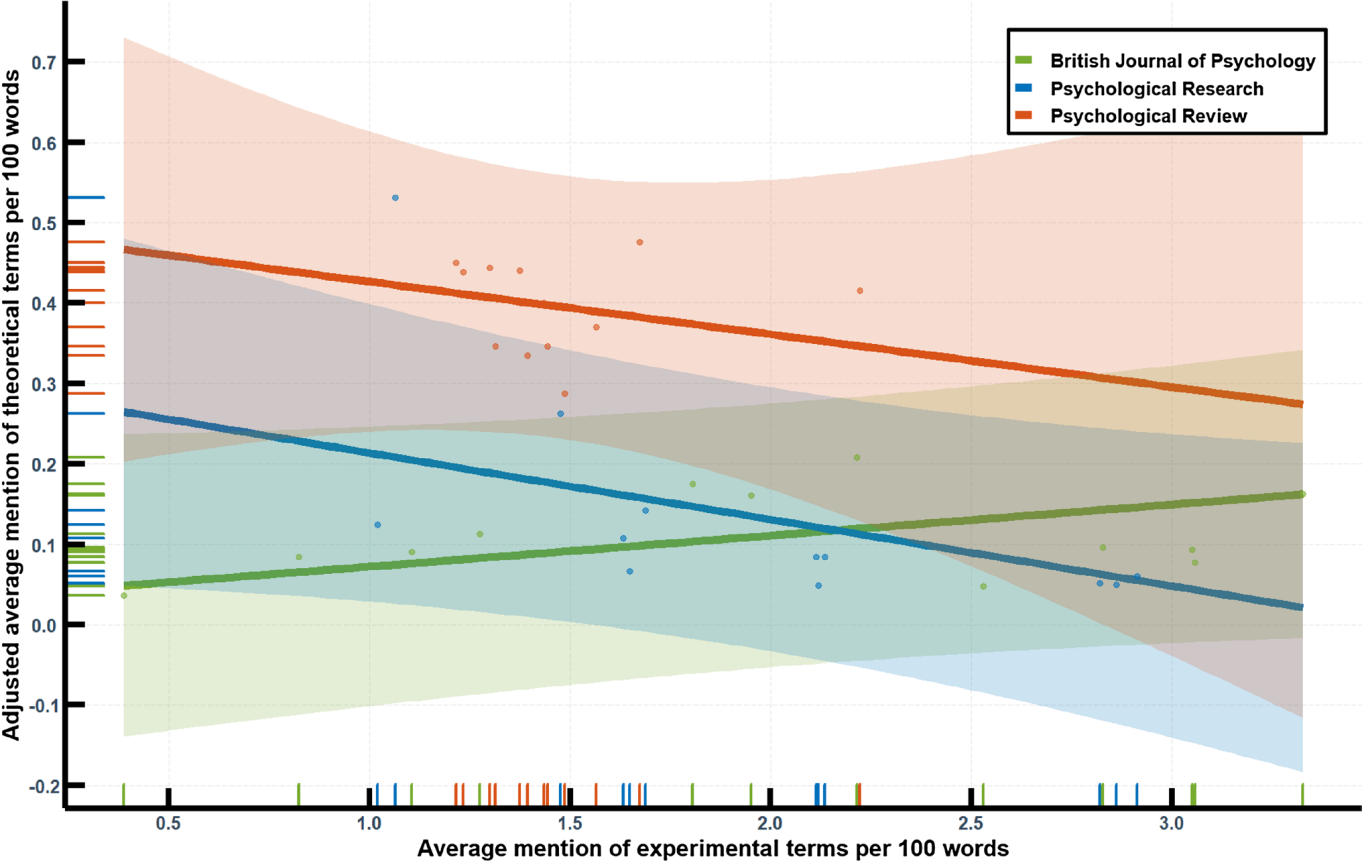
Interaction between experimental terminology and journal in a regression analysis of theoretical vocabulary. The diagram includes 95% prediction intervals, a scatter plot of the (adjusted) data points, as well as a rug plot to increase visibility of the data

Similarly to the first comparative analysis, the context of theoretical and empirical vocabulary invites the question if the development in *Psychological Research* assimilates the general trend or sticks out in the broader context of the discipline. Figure 7 shows an identical design as has been used in the first word frequency analysis, an exploratory regression since at least two journals under considerations show relevant, although not high correlations of the respective frequencies (*Psychological Research*, *r*(10) = -0.63, British Journal of Psychology, *r*(10) = 0.25, Psychological Review, *r*(10) = 0.02). The model has a sufficient fit, *R*^2^ = 0.80, *F*(6,29) = 18, *p* < 0.001. However, unlike the first analysis, time cannot account for the majority of the variance. Instead, the other effects, including the interaction effect of the British Journal of Psychology and experimental terminology provides better explanation.

Although these findings are not apodictic, it becomes clear that the shift from perceptual to cognitive psychology is not analogous to that of theoretical and experimental psychology. The publications in both journals, *Psychological Research* and Psychological Review, suggest that an increase in experimental terminology has come along with a reduction of theoretical terminology while the history of the British Journal of Psychology seems to demonstrate that this is not necessary. Against this background it can be seen that the discursive transformation concerning theoretical psychology is not as linear and homogeneous as the cognitive turn. It calls for more detailed investigation (Table 4).

**Table 4:** The 20 most frequent terms in the abstracts of published articles over period of service for each of the sixth editors-in-chief. Absolute frequency in brackets

Freeman	Scheerer	Heuer	Engelkamp	Frensch	Hommel
1974–79	1979–91	1991–95	1995–01	2001–09	2009–
Visual (77)	Experiment (256)	Experiment (168)	Experiment (206)	Task (492)	Task (1476)
Stimulus (60)	Subjects (225)	Effect (146)	Task (191)	Effect (340)	Participants (974)
Experiment (45)	Experiments (187)	Subjects (137)	Effect (165)	Response (318)	Effect (778)
Test (41)	Task (163)	Experiments (118)	Experiments (161)	Experiment (303)	Experiment (658)
Experiments (39)	Information (158)	Task (105)	Memory (139)	Effects (301)	Study (607)
Subjects (39)	Visual (153)	Stimulus (104)	Subjects (133)	Participants (237)	Effects (583)
Stimuli (38)	Effect (146)	Memory (87)	Effects (117)	Target (235)	Response (573)
Model (37)	Words (135)	Effects (79)	Response (115)	Experiments (216)	Performance (570)
Effect (34)	Stimulus (129)	Target (76)	Stimulus (111)	Visual (212)	Control (536)
Time (34)	Memory (125)	Test (76)	Target (111)	Stimulus (210)	Tasks (532)
Task (33)	Recall (117)	Performance (71)	Performance (106)	Spatial (203)	Visual (451)
Memory (27)	Tasks (105)	Information (70)	Model (103)	Learning (196)	Stimuli (449)
Data (26)	Processing (101)	Response (65)	Test (102)	Study (176)	Memory (447)
Processes (24)	Effects (97)	Study (63)	Visual (90)	Attention (174)	Processing (446)
Conditions (23)	Conditions (95)	Visual (62)	Study (88)	Action (159)	Action (431)
Response (23)	Performance (95)	Simon (60)	Learning (80)	Stimuli (159)	Attention (425)
Theory (23)	Stimuli (93)	Attention (59)	Information (77)	Performance (156)	Cognitive (421)
Experimental (22)	Model (92)	Spatial (57)	Processing (77)	Location (154)	Experiments (412)
Words (21)	Time (82)	Perceptual (56)	Stimuli (76)	Processing (149)	Spatial (375)
Constant (18)	Word (81)	Model (53)	Time (76)	Time (145)	Findings (366)

The findings of the second frequency analysis are crucial for the research question we have posed. Hence, it is worthwhile to give them a closer look. Table 4 shows the 20 most frequent terms in the texts of abstracts of published articles over period of service for each of the sixth editors-in-chief. The terms were obtained by exclusion of non-psychological or non-scientific terms, such as "either", "that", and "with". To some degree, these lists represent the editorial profile of the chief editors. The table gives a detailed look into the discursive transformation in the journal’s history.

The term ‘theory’ does only appear among the first 20 keywords in the period of service of Robert B. Freeman, the first editor-in-chief. Afterwards it does not only disappear from the 20 most frequent terms, it also loses further ranks. During the service of Scheerer it was ranked 34th with 63 mentions, for Heuer 67th (24 mentions), for Engelkamp 76th (32 mentions), for Frensch 217th (28 mentions), and for Hommel so far 254th with 72 mentions. This means that the description of articles has become more and more independent of vocabulary that makes references to theories – a clear indication for the demise of theoretical reflection in the discourse.

It would be an exaggeration to consider the different bibliometric analyses that we have conducted as conspicuous proof of discursive transformations. They are abstract traces which demand more detailed interpretation, be it from the standpoint of linguistics or theoretical psychology. Yet, the findings point in a similar direction: There have been discursive transformations with patterns in form and content. These transformations are multi-layered and concern standardisation of research practise, the transition from continental to the anglophone style, but also the onset and spread of cognitivism.

Regarding the question of theory, the loss of relevance of related terms as well as the increasing presence of vocabulary that describes experimentation coincides with the claim that the psychological discourse has moved from holism to mereological particularism. The precise description of experimental techniques and their interpretation has priority while the integration into the broader context of theory does not. Yet, bibliographic data can allude to content, but it is not identical with it. Hence, the last section will explore the nature of theoretical discourse in *Psychological Research* to complement our bibliographic analysis.

## The relevance of theoretical discourse

Our bibliographic data indicate that the psychological research customs have changed over the course of the last century. This is bound to be surprising. Yet, understanding the nature of this change is necessary to grasp the relation between current and past research. Theoretical psychology and theory in psychology are the focus of our investigation, but the notion of theory is ample. Quantitative data alone cannot elucidate its meaning for the given context. Thus, we wish to present an example from the journal’s history which demonstrates how theory has been incorporated into psychological research in the past.

The disadvantage of examples from the past is their anachronistic nature. This means that it is not trivial to draw conclusions from it. Suggesting a return to almost forgotten rhetoric and structure of argumentation would be nothing short of naïve nostalgia or even a reactionary attitude. What is needed is a synthesis. When research contains critical reflection of its own past and historic conditions, it can guide its progress and anticipate its own direction. ‘Progress’ can only be identified as improvement instead of merely moving onwards due to such critical reflection. Hence, our look into a past instance of theoretical discourse is not meant to serve as an example for the future but as a reminder of the epistemological framework for investigations. This reminder hopefully offers the possibility to do psychological research in awareness of and responsibility for its theoretical foundation. For example, historical comparison highlights the differences in the level of description and explanation (cf. Bechtel, 1994).

The choice of example is not difficult. The journal’s history provides a plethora of contributions that display elaborate engagement with theory. Especially in the first decades, the development of consistent theory has been a common interest among the authors of the journal who—to a large extent—have been representatives of the so-called Berlin school of Gestalt psychology. This is not a minor detail for the analysis of theoretical psychology in the journal’s history. On the contrary, the social coherence of a scientific movement has facilitated the cooperative effort to develop complex theories since the many individual publications were partaking in a conjoint discourse due to institutional background.

### A paradigmatic example

In the following, we will discuss Kurt Koffka’s *Some Remarks on the Theory of Colour Constancy* from 1932. Koffka left Germany as early as 1926 to take up a professorship at the University of Wisconsin, where he also wrote his *Principles of Gestalt Psychology* (1935). He and his students Fritz Heider, Alexander Mintz, Tamara Dembo and Eugenia Hanfmann made a decisive contribution to the spread of Gestalt psychology in the USA (see Mandler, 2007). His article is paradigmatic in a couple of aspects.

First, it is part of a remarkable series of publications called ‘Contributions to psychology of shape (*Gestalt*)’. This series has been documented by Guss (2019). It comprises 26 texts which have been published between 1913 (another contribution by Koffka, published in the *Zeitschrift für Psychologie*) and 1937 (the last part of the series was written by Eugenia Hanfmann and published in *Psychological Research*). The series has been edited by Koffka himself whereas further series were overlooked by different editors of the journal. Guss (2019) mentions five separate series.

Second, Koffka’s text has a sizeable length of 26 pages. It is noteworthy, however, that the first decades of the journal have seen several longer publications and, among them, entire PhD theses. Koffka’s *Remarks* are a regular publication that presents empirical results as well as an extensive discussion of the results in the context of different theoretical traditions of the time, namely the attempted explanations of the phenomenon of ‘Colour Constancy’ by Katz, Gelb, Bühler, and Jaensch as well as their disciples. Unlike current publications in the APA format, the discussion is not formally separated from the reported results. Instead, the article begins by discussing theory and continues to do so until its last patch. The article consists of seven sections, followed by a summary in English and German.

Third, Koffka’s text is not confined to theory. He describes and discusses several investigations, albeit in the Gestaltist tradition and with the quantitative methodology of his time, lacking sample-based and randomized measurement. On the contrary, his approach relies on the intuitive validity of manipulation which reflects in a characteristic expression when he speaks of an “observation which I made long ago and which can be repeated every day by everyone” (Koffka, 1932, 332). Nevertheless, the article comprises empirical reports, descriptions of experimental setups, logic arguments, presentations of other researchers’ work, and even mathematical formulae. Hence, the text is able to represent the state of art of its day in several regards.

Koffka’s research interest is founded in a philosophical concern of the late nineteenth and early twentieth century that can already be found in the school of Graz, for example in Alexius Meinong (cf. Meinong, 1914, 349). He describes the problem with the following question: “why the objects of our environment change so little in colour despite the wide variations in the intensity and composition of the light rays reflected by them” (Koffka, 1932, 339). Fundamentally speaking, this question concerns the relation between visual stimuli and the constitution of the phenomenal world. Yet, we do not wish to problematize the subject matter on this occasion. Rather, we take a look at the place of theory in the article.

Right in the beginning, Koffka mentions that the problem of colour constancy is responsible for “stirring up a good deal of theoretical discussion” (ibid.). Koffka, then, proceeds to engage with the different theoretical alternatives for the explanation of constancy. He critically examines the arguments put forth by Katz against Mintz. The analysis is logical and does not draw on empirical data. This changes in the second section. Koffka emphasizes: “I believe that at the moment we need some really concrete hypotheses which will force the experimental work done in our field into definite channels by which true theoretical decisions can be reached” (ibid., 332). From a contemporary perspective, it must appear seldom that empirical psychological research requires such justification. Yet, Koffka’s reasoning is an expression of the roots of psychology in philosophy where empirical arguments have been used to tackle problems that could otherwise also be solved logically or even speculatively.

Koffka’s presentation of his empirical investigation is not exhaustively summarized by the report of procedures or the experimental setup. He continuously develops his terminology. For example, he proposes the “phenomenological or psychophysical concept of level” (ibid., 336). When he says ‘phenomenological’, he primarily refers to the goal of precise observation. Thus, Koffka attempts to encounter the best description for the phenomenon. This can be seen in his meta-reflections, such as: “My own observation is that in many cases we see illumination, although I feel certain that […]”; or “Perhaps we might say that illumination is rather ‘felt’ than ‘perceived’” (ibid, 351).

Ultimately, Koffka is in search of an *experimentum crucis*, or, as he says, a “truly crucial experiment” (ibid., 348) that can corroborate the theories in question. This epistemological principle guides his line of argument when he says: “It is clear that this experiment excludes an explanation by contrast” (ibid., 341). Accordingly, he reflects on the plain of epistemology and the conditions for conclusions from empirical data. One example for this kind of argument is his comment on the controversy between Jaensch and Katz: “It is true, Jaensch did not sufficiently distinguish between the colour of illumination as influencing the colour of the appearing objects and as being a datum in its own right” (ibid., 349). This is a conceptual reflection. It does rely on empirical observations, but the scientist draws on logical reasoning to determine the status of the respective theory.

Importantly, Koffka does not only interpret the available empirical data, but he also discusses the psychological framework for this interpretation. In a telling passage he says: “We have to take into account the organization of the whole field, not only of that part in which our experiment takes place” (ibid., 343). This way he makes himself accountable for the conclusions he draws from his experimentation. He questions the presuppositions of his research and obtains an epistemological standpoint, namely holism which stands in contrasts with elementarism.

Overall, theory is not a mere concomitant of Koffka’s article, developing theory is its main purpose. The presented empirical results serve as logical arguments that are supposed to mediate between competing theoretical approaches. In stark contrast to the contemporary role of theory in psychological research, Koffka does not aim to develop a model whose purpose is the precise representation of measurement alone. Rather, his theoretical contemplations are meant to answer to the available alternatives, to discuss the validity of observations, and to be accountable for the underlying epistemological problem of colour constancy.

### Theoretical psychology—perspectives for the future

Much has changed in the standards and customs of psychological research since Koffka’s text has been published in the early 1930s. One will not find many traces neither of argumentation nor of content in recent research. This constitutes descriptive evidence for a fundamental discursive transformation. In the last section we turn to its explanation as well as the evaluation of its significance for the discipline as a whole.

Chisholm (1973) proposes a useful distinction that can help determining the essential discursive transformation within psychology. He uses the opposing terms “methodism” and conceptual “particularism” (in contrast to *mereological* particularism, see above) to address different scientific attitudes which both generalize a certain epistemic faculty to contradict skepticism. These attitudes have originally been employed on a logical plain to tackle the ‘problem of the criterion’. This epistemological problem concerns the valid sources of knowledge (cf. Fumerton, 2008). Generally speaking, conceptual particularists rely on (particular) beliefs as the foundation of knowledge, Methodists, on the other hand, rely on methods. Nevertheless, the terms have also been used to distinguish research styles in psychology (Münch, 2002).

Chisholm’s notion of ‘methodism’ can be used to characterize the contemporary status of psychological epistemology. Whereas Koffka discussed the very foundations of psychological insight in his text, comparable issues are, so to say, outsourced to a separate methodological plain today. This plain is the construction and development of (quantitative) methods of experimentation and interpretation. The reason for this difference can be found in ‘methodism’, i.e., in the scientific attitude of trusting the functionality of experimental methods. Differently put, the assumption of a continuous turn towards methodism in the last century can account for the diminishing relevance of theory as a discursive transformation.

More concretely speaking, psychology can either investigate the methodological conditions of its empirical conclusions itself or it can leave this foundational issue outside of empirical research (methodism). While the prior is true for Koffka, the latter seems to be predominant in current research. This development seems to be natural considering the substantial increase in complexity and diversity of methods within experimental science: Unlike 1930s methodology, nowadays the technological and mathematical substructure of psychology is too ample to be made explicit every time results are reported.

If this explanation is accurate, one consecutive question might be what has caused the transformation. Several perspectives can be taken into account: Sociology of science, for example, might entertain the hypothesis that European psychology has been influenced by American approaches over the course of the twentieth century and therefore by a more pragmatist philosophy of science (cf. Métraux, 1985). In a similar vein, history of psychology could invoke the shift from the Gestaltist paradigm to so-called cognitive sciences (Ash, 1998). Instead of deliberating possible causes for the development of the discipline, we wish to finish our investigation by asking what the value of theoretical psychology is or could be today.

The notion of ‘methodism’ which we have employed to explain the discursive transformation in psychology over the course of the last century might mislead one to assume a conceptual opposition of theoretical deliberation on the one side and methodological rigor on the other. It is only partly true that the availability of an extensive body of experimental methods has made theoretical reflection unnecessary in psychology. We want to claim this issue as a central problem of theory in contemporary psychology: Theoretical discourse is necessary for the reflection of epistemological foundations. This is contradictory to the tendency to rely on complex methods for empirical research because they presuppose epistemological assumptions as constitutive for their procedures. Dietrich Dörner has articulated this issue: “We must not succumb to ‘methodism,’ however, choosing an action only because it has often worked before” (Dörner, 1989, 198). This ‘problem of methodism’ calls for a solution that offers a synthesis between empirical methodology and theoretical discourse.

What necessitates this synthesis are the epistemological difficulties of experimental psychology which cannot be tackled by empirical research alone, for example the problem of disciplinary integration (Bender, 2022) or the choice of normative principles (Fiedler et al., 2022). These issues cannot be solved outside of the psychological discourse, for example in philosophy. They call for a further discursive transformation which leads back to theory. Theoretical discourse, either in the form of 1. theoretical psychology or of 2. theory about psychological constructs and models, can serve to complement quantitative methodology in different forms.

1. Theoretical psychology reflects on the epistemological and ontological background of experimentation. For example, theoretical psychology coordinates individual experimental paradigms and broader issues, such as the ‘hard problem of consciousness’ or the ‘mind–body problem’, insofar as they matter for the outline of the investigation. Koffka gives an example for the relevance of this discourse when he debates how holism affects his empirical hypotheses. A paradigmatic contemporary case for this kind of problem would be so-called eliminative materialism as a framework for neuroscience (Churchland, 1995; Rorty, 1970) and its critique (Slagle, 2020). The controversy concerns the role of folk psychological concepts in the understanding of cognitive processes. Not only do many, if not all psychological investigations correspond to it due to their background, their results can also contribute to advancing the fundamental discussion. Hence, the integration of theoretical psychology would enable a two-way dialogue.

2. It would be an exaggeration to claim that theory did not play a role in the contemporary discourse. Yet, in light of the so-called replication crisis, Oberauer and Lewandowsky (2019) make a case for a corresponding ‘theory crisis’. Their proposal of a solution focusses on the idea of “formalizing theories as computational models” (Oberauer & Lewandowsky, 2019, 1596). What is the field of research that can assess the value of this suggestion? Psychology hosts different forms of theories on the object level of models and constructs, and formal models are but an example. The way psychologists think about their subject matter depends on the customs of theorizing, for example their standard of making explanations (Bechtel & Wright, 2019). The quality of this theorizing should not be left to chance, but only a meta-discourse which is well-structured and rich in perspectives can provide security. The decade-long series of publications called ‘Contributions to psychology of shape’ can give a good example for the necessary effort that needs to be put into theorizing. Koffka’s investigation is integrated into a network of competing theories which brings different epistemological standpoints into a dialogue.

According to Van Hezewijk (2000), theoretical psychology should help “to (re)construct the non-empirical (metaphysical) claims implied by empirical hypotheses” (van Hezewijk, 2000, 103/104). Therefore, it is necessary to focus on ‘Why-questions’ instead of ‘What-is-questions’ in psychological research. As a consequence, theoretical psychology “can help to find objective procedures for deciding between knowledge claims of both an empirical and non-empirical (metaphysical) nature” (ibid., 104). However, only a vivid theoretical discourse can provide the critical basis to evaluate such knowledge claims. This discourse is not entirely absent in contemporary research, but it should be prioritized and perceived as a primary scientific responsibility in the discipline.

Theoretical psychology and a multi-facetted theoretical discourse play an essential role in the growth and progress of the discipline. However, a discourse of this kind cannot be constructed because it requires expertise. It is a question of scientific culture to maintain a sufficient level of conceptual reflection. We as psychologists need to keep a continuous theoretical dialogue alive that provides orientation and conceptual alternatives. Our investigation has shown that there is reason to doubt whether the last decades have seen sufficient effort in this regard. What might be needed is a further discursive transformation which re-integrated extensive theoretical debate into the culture of our discipline. Nonetheless, throughout the last century, the editors of *Psychological Research* have demonstrated that they are aware of the responsibility for theoretical discourse. They made contributions to the respective debates (e.g., Hommel, 2019) and promoted the exchange about theory, based on interdisciplinary and historical reference. Hence, the journal promises to be a platform and forerunner of future discursive transformations.

Gigerenzer (2010) rightfully suggests that theory construction and theory integration belong together, and that research should focus on psychological subject matters and not on sub-disciplines to gain an interdisciplinary perspective that enables the integration of theories. This responsibility cannot be fulfilled by the construction of models alone. What is required, is a research culture of theoretical debate and controversy (cf. Wendt & Funke, 2022): Theories may not only serve an instrumental purpose or be reducible to models for empirical predictions. Debate culture can be seen as a type of solidarity among scientists who recognize competing positions because they realize that overcoming one’s limitations requires triangulation. However, this does not imply neutral coexistence, but earnest dialogue in which different perspectives compete for the adequate description and explanation of a subject matter. The culture of debate is the manifestation of pluralism.
